# The spatial–temporal variations and influencing factors of COVID-19 case fatality rate: a worldwide study in 30 countries from February 2021 to May 2022

**DOI:** 10.1017/S0950268824000852

**Published:** 2024-10-17

**Authors:** Jing Zhao, Xing Huang, Xing Li, Bing Li, Zuhua Rong, Xu Huang, Ruiqi Ren, Dan Li, Chao Li, Qun Li, Jianpeng Xiao, Guoqing Shi

**Affiliations:** 1 National Key Laboratory of Intelligent Tracking and Forecasting for Infectious Diseases, Chinese Center for Disease Control and Prevention, Beijing, China; 2 School of Public Health, Southern Medical University, Guangzhou, China; 3 Guangdong Provincial Institute of Public Health, Guangdong Provincial Center for Disease Control and Prevention, Guangzhou, China; 4 Chinese Center for Disease Control and Prevention, Beijing, China

**Keywords:** case fatality rate, COVID-19, influencing factors, spatial–temporal changes, public health interventions, meta-analysis

## Abstract

To evaluate the variations in COVID-19 case fatality rates (CFRs) across different regions and waves, and the impact of public health interventions, social and economic characteristics, and demographic factors on COVID-19 CFRs, we collected data from 30 countries with the highest incidence rate in three waves. We summarized the CFRs of different countries and continents in each wave through meta-analysis. Spearman’s correlation and multiple linear regression were employed to estimate the correlation between influencing factors and reduction rates of CFRs. Significant differences in CFRs were observed among different regions during the three waves (*P* < 0.001). An association was found between the changes in fully vaccinated rates (*r*_*s*_ = 0.41), population density (*r*_*s*_ = 0.43), the proportion of individuals over 65 years old (*r*_*s*_ = 0.43), and the reduction rates of case fatality rate. Compared to Wave 1, the reduction rates in Wave 2 were associated with population density (*β* = 0.19, 95%*CI*: 0.05–0.33) and smoking rates (*β* = −4.66, 95%*CI*: −8.98 – −0.33), while in Wave 3 it was associated with booster vaccine rates (*β* = 0.60, 95%*CI*: 0.11–1.09) and hospital beds per thousand people (*β* = 4.15, 95%*CI*: 1.41–6.89). These findings suggest that the COVID-19 CFRs varied across different countries and waves, and promoting booster vaccinations, increasing hospital bed capacity, and implementing tobacco control measures can help reduce CFRs.

## Introduction

As of August 2023, more than 770 million people have been infected by COVID-19, resulting in over 6.9 million deaths worldwide [1]. COVID-19 has rapidly spread globally and multiple waves of infection have occurred [2]. From a spatial perspective, the case fatality rates (CFRs) for COVID-19 exhibited variability across different regions. At the national level, early studies revealed significant variability in the CFRs among countries, with the COVID-19 CFRs ranging from 0% to over 20% [3]. From a temporal perspective, the CFRs of COVID-19 showed variations across different waves of the epidemic, even within the same geographical location. For example, the CFR decreased over time in Regensburg, Germany from March 2020 to January 2022, most prominently from September 2021 to January 2022 [4]. Considering the underlying factors for temporal and spatial disparities in COVID-19 CFR is crucial, so as to find out the characteristics of the pandemic.

Several elements must be considered, and the emergence of virus strains may be one of the important factors. Unlike the Alpha, Beta, and Gamma variants, the Delta variant was believed to have a higher level of transmissibility and pose a greater risk of hospitalization and mortality [5]. It has been observed that the Omicron variant exhibited a high transmission rate while displaying relatively low lethality [6], which may contribute to a lower CFR to a certain extent.

Regarding other factors that influence COVID-19 CFRs, some previous research has indicated that the implementation of public health interventions, such as COVID-19 testing and vaccination [7], may be associated with a reduction in CFRs. Additionally, social and economic characteristics of a country, including population density [8], economic development, and access to medical resources [9], have been shown to impact COVID-19 risk. Demographic characteristics, such as age [10], common chronic diseases [11], and smoking habits [12], are also believed to play a role in determining an individual’s risk of severe illness or death from COVID-19. Even though the COVID-19 emergency phase has been ended and transitioned to longer-term disease management, the impact of the evolving pandemic and the promotion of prevention measures, particularly COVID-19 vaccination, on changes in CFRs across different waves remains uncertain.

In this paper, we examined the temporal and spatial variations of COVID-19 CFRs and attempted to identify key factors possibly explaining the variability in CFRs across countries during three waves of the COVID-19 epidemic (22 February to 30 May 2021; 1 July to 22 October; 22 December 2021 to 22 May 2022).

## Methods

### Data sources

We selected three global epidemic waves since 2021 as the period to investigate the temporal and spatial variations of COVID-19 CFRs following vaccination and the implementation of COVID-19 prevention and control measures. The dates of the three waves were defined as 22 February to 30 May 2021 (Wave 1), 1 July to 22 October 2021 (Wave 2), and 22 December 2021 to 22 May 2022 (Wave 3) (Supplementary Figure S1). Based on the two lowest points of each wave, the dates for each wave were determined. During our study period, the global COVID-19 fully vaccinated rate increased from 0.5% to 59.0%. Moreover, these waves provide valuable insights into the epidemic periods of various COVID-19 variants, including Alpha (Wave 1), Delta (Wave 2), and Omicron (Wave 3) [13].

Even though a prior wave occurred from October 2020 to February 2021, the global vaccination campaign began in late 2020 and was largely completed by early 2022. In the early stage of implementation, the distribution of vaccine supplies varied widely among countries, a large number of countries even had no access to vaccines, which would introduce uncertainty to the evaluation of vaccination effects. Therefore, data from the period spanning October 2020 to February 2021 were excluded from this study due to uneven vaccine accessibility and a lack of spatial comparability between countries during that period.

**Figure 1.**
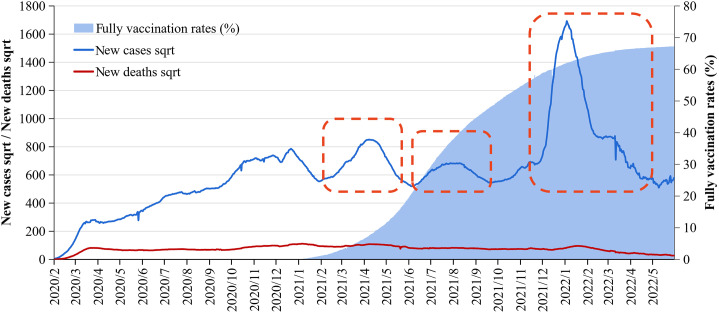
The daily cases, deaths, and fully vaccinated rates of 30 countries from 22 February 2020 to 22 June 2022.

Based on their significant incidence of the initial wave of the COVID-19 epidemic and comprehensive case reports, 30 countries with the highest COVID-19 incidence during Wave 1 were selected. During the study period, these countries accounted for 78% of global COVID-19 cases, and similar epidemic patterns were observed throughout all three waves (Figure 1, Supplementary Figure S2). The potential influencing factors examined in this study included public health interventions (changes in the total tests per thousand people, the stringency index, the fully vaccinated rates, and the booster vaccination rates in Wave 3), social and economic characteristics of each country (GDP, human development index, and hospital beds per thousand people), and demographic factors (the proportion of individuals over 65 years old, diabetes prevalence, obesity rates, and smoking rates). Notably, the data on some indicators may not be regularly updated, and according to the open database, no significant annual relative changes were found. Therefore, we posit that these influencing factors remained relatively stable throughout the study period. Consequently, except for vaccination rates (including fully vaccinated rates and booster vaccination rates), total tests, and stringency index, all other factors were based on the latest annual data available for each country.

All the data we used in this study were extracted from Our World in Data [14] on 3 August 2022.

### Statistical analyses

The COVID-19 CFR in each country was calculated as the ratio of the total deaths to the number of patients during each wave. It is important to note that there is a time lag between the time of infection and death, which has been documented by previous studies [15]. By considering the lag effect on mortality rates and utilizing global infection and death data, the death count of COVID-19 was calculated with a lag of 12 days. For instance, the CFR of India in Wave 1 was the ratio of the total number of COVID-19 deaths occurring between 6 March and 11 June 2021, to the number of COVID-19 patients present between 22 February and 30 May 2021.

A meta-analysis was used to explore the variations in CFRs among countries and continents during each wave. Besides, Spearman’s correlation analysis and multiple linear regression were utilized to estimate the association between the reduction rates of COVID-19 CFRs across different waves and these potential factors.

The potential covariates incorporated in each model were derived from a synthesis of previous studies [16, 17]. The results of correlation analysis and collinearity between different variables are presented in the appendix (Supplementary Figures S3–S5). Given the paramount significance of COVID-19 vaccination rates, we prioritized the exclusion of factors exhibiting significant collinearity with vaccination rates in all three regression models. When certain collinear variables were unavoidably included in the model, we sequentially added them one by one and evaluated the model fit; the variable producing the best fit was retained in the model.

Therefore, in regression model 1, the change in total tests per thousand people, the proportion of individuals over 65 years old, GDP, and human development index were excluded due to their high collinearity with fully vaccinated rates. In regression model 2, the changes in stringency index, total tests per thousand people, fully vaccinated rates, the proportion of individuals over 65 years old, obesity prevalence, and human development index were not incorporated. Similarly, the change in stringency index, fully vaccinated rates, and the proportion of individuals over 65 years old, GDP, and smoking rates were not considered in regression model 3.

The multiple linear regression equation estimated reduction rates of CFR as follows:
(1)

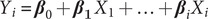

In Eq. (1), ***Y***_***i***_ represented the reduction rates of CFR, ***β***_***0***_ was the intercept, and ***X***_***1***_ to ***X***_***i***_ were the independent variables for each reduction rate of CFR.

For sensitivity analysis, the date of each wave was expanded by 14 days (with both the commencement and termination dates of each wave being extended by 7 days) to assess whether the date we defined would have any impact on the results.

Given the negative values of the reduction rates of CFR, a transformation was applied to the dependent variable in Spearman’s correlation analysis and multiple linear regression (by adding 100 to the reduction rates of CFR). In this study, a P value <0.05 was considered statistically significant. Our analyses were conducted with R software (R 4.2.1).

## Results

### The COVID-19 CFRs in 30 countries during three COVID-19 waves

Table 1 presented the descriptive results regarding the cases and deaths of COVID-19 in 30 countries during three epidemic waves.

**Table 1.** The temporal and spatial variations of COVID-19 CFRs in 30 countries during three waves

Countries	Wave 1 (February 22–May 30, 2021)				Wave 2 (July 1–October 22, 2021)					Wave 3 (December 22, 2021 - May 22, 2022)					
	Cases (10^3^)	Daily cases (10^3^)	Deaths (10^3^)	CFR (%)	Cases (10^3^)	Daily cases (10^3^)	Deaths (10^3^)	CFR (%)	Reduction 1^a^(%)	Cases (10^3^)	Daily cases (10^3^)	Deaths (10^3^)	CFR (%)	Reduction 2^b^ (%)	Reduction 3^c^ (%)
India	16,878.4	172.2	197.9	1.17	3,685.0	32.3	47.8	1.30	−10.57	8,370.4	55.1	42.6	0.51	56.57	60.72
Brazil	6,274.2	64.0	217.8	3.47	2,947.7	25.9	72.1	2.45	29.52	8,535.2	56.2	47.5	0.56	83.96	77.24
United States	5,039.5	51.4	72.3	1.44	11,577.7	101.6	143.0	1.24	13.93	31,757.0	208.9	180.5	0.57	60.41	54.00
Turkey	2,589.5	26.4	19.6	0.76	2,341.7	20.5	20.7	0.89	−17.19	5,833.6	38.4	16.2	0.28	63.30	68.69
France	2,398.4	24.5	21.8	0.91	1,332.4	11.7	6.4	0.48	47.47	20,583.4	135.4	24.2	0.12	87.08	75.40
Argentina	1,662.5	17.0	31.1	1.87	786.0	6.9	16.4	2.08	−11.44	3,719.8	24.5	11.6	0.31	83.25	84.97
Italy	1,394.2	14.2	27.3	1.96	468.9	4.1	4.4	0.93	52.55	11,756.8	77.3	29.0	0.25	87.37	73.39
Iran	1,310.9	13.4	21.1	1.61	2,614.7	22.9	40.4	1.55	3.88	1,051.9	6.9	9.6	0.91	43.14	40.85
Germany	1,284.4	13.1	17.8	1.38	688.7	6.0	4.6	0.66	52.02	19,165.6	126.1	27.1	0.14	89.79	78.71
Poland	1,228.7	12.5	29.3	2.38	76.2	0.7	1.8	2.43	−1.75	2005.2	13.2	18.7	0.93	60.81	61.48
Colombia	1,133.4	11.6	33.6	2.97	717.0	6.3	13.5	1.88	36.51	986.4	6.5	9.8	0.99	66.5	47.24
Ukraine	903.4	9.2	25.2	2.79	526.2	4.6	18.1	3.44	−23.28	1,439.1	9.5	13.0	0.90	67.72	73.82
Russia	865.2	8.8	35.9	4.15	2,532.7	22.2	94.4	3.73	10.27	7,907.0	52.0	66.5	0.84	79.74	77.42
Peru	664.2	6.8	61.4	9.25	134.7	1.2	5.7	4.23	54.23	1,308.3	8.6	10.4	0.80	91.37	81.16
Philippines	653.1	6.7	9.8	1.51	1,321.8	11.6	17.3	1.31	13.13	851.6	5.6	8.9	1.04	30.88	20.43
Spain	589.0	6.0	9.3	1.58	1,173.9	10.3	6.4	0.54	65.62	6,589.7	43.4	17.1	0.26	83.61	52.33
Netherlands	583.2	6.0	1.9	0.33	385.7	3.4	0.7	0.18	43.44	5,053.7	33.2	1.4	0.03	91.73	85.38
Chile	566.6	5.8	9.3	1.65	117.5	1.0	4.0	3.39	−105.69	1842.2	12.1	7.3	0.40	75.94	88.30
Canada	529.9	5.4	3.7	0.69	277.9	2.4	2.6	0.94	−35.79	1926.0	12.7	11.2	0.58	15.80	37.99
Iraq	521.8	5.3	3.1	0.59	688.7	6.0	5.6	0.81	−36.71	235.7	1.6	1.0	0.44	25.36	45.40
Indonesia	521.1	5.3	15.2	2.92	2034.7	17.8	75.2	3.70	−26.61	1791.3	11.8	12.5	0.70	76.11	81.13
Czechia	506.0	5.2	8.7	1.71	52.5	0.5	0.5	0.93	45.5	1,511.1	9.9	4.0	0.27	84.39	71.35
Sweden	437.3	4.5	1.6	0.36	74.6	0.7	0.4	0.53	−49.66	1,238.4	8.1	3.7	0.30	16.26	44.05
Hungary	398.4	4.1	14.1	3.55	33.1	0.3	0.9	2.75	22.53	681.0	4.5	7.1	1.04	70.55	61.99
Mexico	367.9	3.8	35.0	9.50	1,247.2	10.9	53.2	4.26	55.11	1815.8	11.9	25.4	1.40	85.28	67.20
Jordan	366.4	3.7	4.7	1.27	94.1	0.8	1.2	1.28	−0.64	650.6	4.3	1.3	0.20	84.04	84.14
United Kingdom	362.1	3.7	3.4	0.95	3,820.8	33.5	12.5	0.33	65.65	9,796.7	64.5	25.9	0.26	72.29	19.34
Pakistan	345.6	3.5	8.4	2.42	307.7	2.7	5.8	1.90	21.72	237.3	1.6	1.4	0.60	75.06	68.14
Japan	315.5	3.2	5.7	1.80	918.3	8.1	3.3	0.36	80.06	6,853.5	45.1	12.3	0.18	90.05	50.13
Belgium	304.2	3.1	2.8	0.93	219.8	1.9	0.8	0.36	61.49	2,111.0	13.9	3.4	0.16	82.76	55.24

Abbreviation: CFR, the case fatality rate of COVID-19.

a The reduction rates of COVID-19 CFRs in Wave 2 compared with the CFRs in Wave 1.

b The reduction rates of COVID-19 CFRs in Wave 3 compared with the CFRs in Wave 1.

c The reduction rates of COVID-19 CFRs in Wave 3 compared with the CFRs in Wave 2.

Restricting to each wave, the CFRs varied across different countries and continents (*P* < 0.001) (Supplementary Figures S6–S11). Specifically, the CFRs ranged from 0.33% (Netherlands) to 9.7% (Mexico), 0.18% (Netherlands) to 4.26% (Mexico), and 0.03% (Netherlands) to 1.40% (Mexico), in Wave 1, Wave 2, and Wave 3, respectively.

Compared to Wave 1, the most substantial reduction rate in Wave 2 was 80.6% (Japan), while 91.73% (Netherlands) in Wave 3. And compared to Wave 2, the most significant decrease in CFR during Wave 3 was observed in Chile, with a reduction rate of 88.3%. Notably, among the 30 countries analysed, CFRs in Wave 2 were lower than those in Wave 1, except for India, Turkey, Argentina, Poland, Ukraine, Chile, Canada, Iraq, Indonesia, Sweden, and Jordan, while Wave 3 exhibited a significant decrease in CFR compared to both Wave 1 and Wave 2.

### The descriptive statistics of influencing factors

Table 2 summarized the variables included in the models. Overall, 16 middle-income countries and 14 high-income countries were included in our study. Throughout the three waves of the epidemic, a gradual increase was revealed both in total tests per thousand people and vaccination rates across all three waves of the epidemic in the 30 countries. This trend was observed to be concomitant with a relaxation in government-imposed restrictions.

**Table 2.** Descriptive statistics of model variables

Category	Variable	Mean	SD	Min	Max
Public health interventions	Changes in total tests per thousand people				
	Wave 2 compared with Wave 1	365.18	456.01	24.84	1919.24
	Wave 3 compared with Wave 1	965.79	1,077.26	63.89	4,889.24
	Wave 3 compared with Wave 2	600.62	656.00	34.44	2,970.01
	Changes in stringency index				
	Wave 2 compared with Wave 1	−15.50	13.88	−42.06	7.57
	Wave 3 compared with Wave 1	−28.98	15.41	−52.55	0.02
	Wave 3 compared with Wave 2	−13.48	8.79	−39.88	5.92
	Changes in fully vaccinated rates (%)				
	Wave 2 compared with Wave 1	32.33	16.98	4.95	59.63
	Wave 3 compared with Wave 1	57.88	14.91	15.28	79.79
	Wave 3 compared with Wave 2	25.56	13.42	6.37	51.07
	Booster vaccine rates^a^ (%)	31.75	20.87	0.38	76.20
Social and economic characteristics of the country	Population density	148.01	140.06	4.04	508.54
	GDP	26,158.73	14,661.92	5,034.71	54,225.45
	Hospital beds per thousand people	3.79	3.02	0.53	13.05
	Human development index	0.828	0.100	0.557	0.947
Demographic characteristics	The proportion of individuals over 65 years old (%)	13.25	6.82	3.19	27.05
	Diabetes prevalence (%)	7.47	2.37	4.28	13.06
	Obesity prevalence (%)	11.44	4.10	3.92	23.93
	Smoking rates (%)	16.03	4.42	3.79	22.40

Abbreviation: SD, standard deviation.

a It was calculated in Wave 3 since most countries have not yet implemented booster vaccination plans during the first two waves.

### The correlation analysis between the three reduction rates of COVID-19 CFR and relative influencing factors

**Figure 2.**
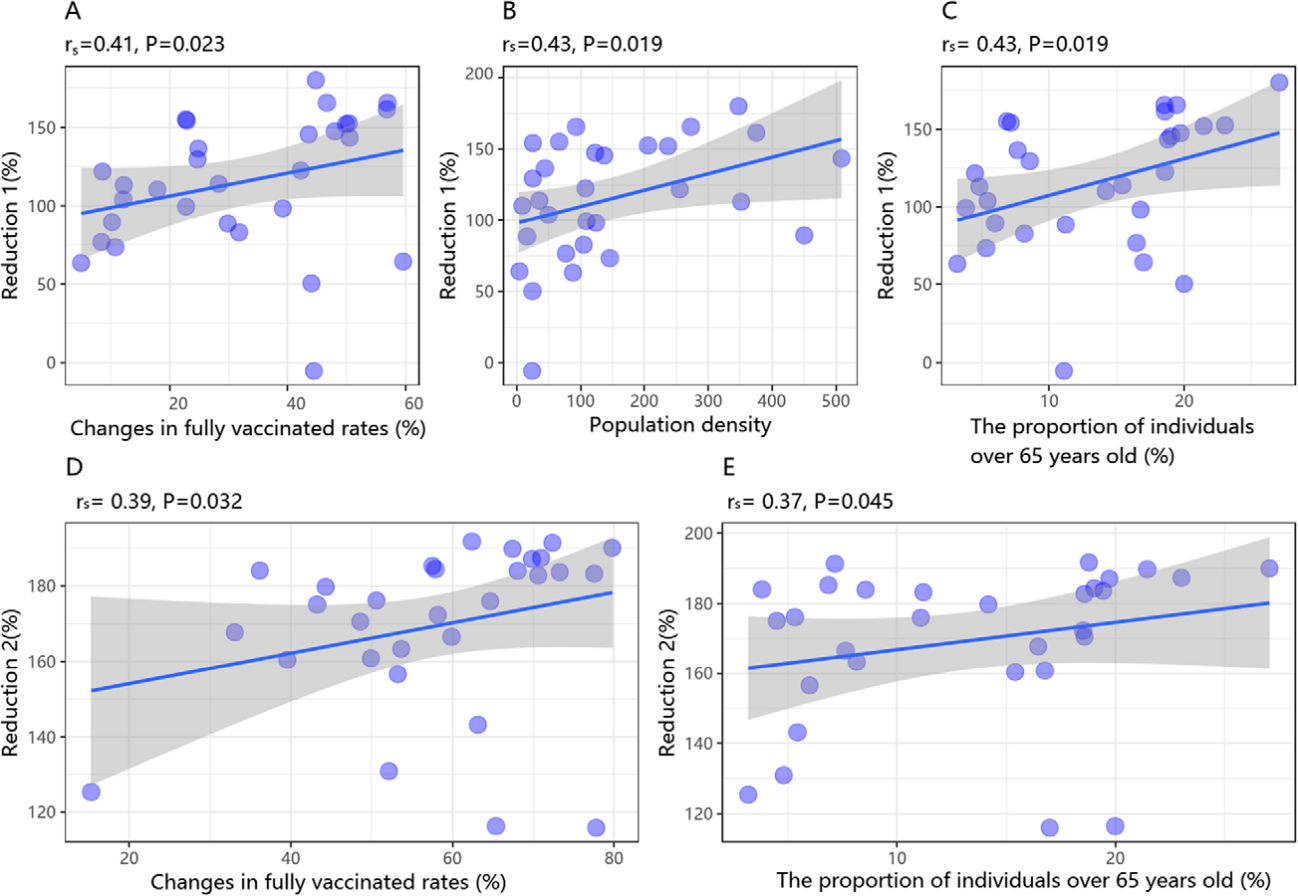
The Spearman’s correlation analysis between the reduction rates of CFRs and change in fully vaccinated rates, population density, and the proportion of the individuals over 65 years old.

In comparison to the CFRs observed in Wave 1, the reduction rates of CFR in Wave 2 (Reduction 1) showed a positive correlation with changes in COVID-19 fully vaccinated rates (*r*_***s***_ = 0.41), population density (*r*_***s***_ = 0.43), and the proportion of individuals over 65 years (*r*_***s***_ = 0.43) (Figure 2).

Similarly, when compared to the CFRs in Wave 1, the reduction rates of CFR in Wave 3 (Reduction 2) were only positively associated with changes in COVID-19 fully vaccinated rates (*r*_***s***_ = 0.39) and the proportion of individuals over 65 years old (*r*_***s***_ = 0.37) (Figure 2 **)**. However, no significant correlation was found between the reduction rates of CFR in Wave 3 and the associated factors as compared to those observed in Wave 2 (Reduction 3) (Table 3).

**Table 3.** The Spearman’s correlation analysis between the reduction rates of CFRs and influencing factors

Category	Variable	Reduction 1^a^ (%)		Reduction 2^b^ (%)		Reduction 3^c^ (%)	
		*r*_*s*_	*P*	*r*_*s*_	*P*	*r*_*s*_	*P*
Public health interventions	Changes in total tests per thousand people	0.16	0.390	0.16	0.396	0.10	0.610
	Changes in stringency index	−0.02	0.897	0.00	0.987	0.02	0.912
	Changes in fully vaccinated rates (%)	0.41	0.023	0.39	0.032	0.11	0.566
	Booster vaccine rates (%)	–	–	0.34	0.06	0.12	0.542
Social and economic characteristics of the country	Population density	0.43	0.019	0.25	0.182	−0.09	0.625
	GDP	0.29	0.125	0.15	0.442	−0.16	0.389
	Hospital beds per thousand people	0.28	0.137	0.34	0.064	0.17	0.363
	Human development index	0.33	0.073	0.21	0.261	−0.11	0.570
Demographic characteristics	The proportion of individuals over 65 years old (%)	0.43	0.019	0.37	0.045	−0.02	0.929
	Diabetes prevalence (%)	−0.33	0.079	−0.29	0.123	0.01	0.994
	Obesity prevalence (%)	−0.29	0.117	−0.16	0.392	0.15	0.418
	Smoking rates (%)	0.04	0.822	−0.07	0.712	−0.15	0.428

a The reduction rates of COVID-19 CFRs in Wave 2 compared with the CFRs in Wave 1.

b The reduction rates of COVID-19 CFRs in Wave 3 compared with the CFRs in Wave 1.

c The reduction rates of COVID-19 CFRs in Wave 3 compared with the CFRs in Wave 2.

### The multiple linear regression analysis between the reduction rates of CFR and relative influencing factors

Table 4 illustrated the associations between influencing factors and Reduction 1 (Model 1), Reduction 2 (Model 2), and Reduction 3 (Model 3). Our analysis revealed that population density (*β* = 0.19, 95%CI: 0.05–0.33) was significantly associated with an increase in the reduction rates of CFR in Wave 2 when compared to Wave 1 while smoking rates demonstrated a noteworthy negative association (*β* = −4.66, 95%CI: −8.98 – −0.33) with the reduction rates. In Model 2, a significant positive correlation was observed between booster vaccine rates (*β* = 0.60, 95%CI: 0.11–1.09) and an increase in the reduction rates of CFR in Wave 3 when compared with Wave 1. Moreover, a higher number of hospital beds per thousand people (*β* = 4.15, 95%CI: 1.41–6.89) was found to be significantly associated with this increase. Additionally, in Model 3, the booster vaccine rates (*β* = 0.67, 95%CI: 0.09–1.24) demonstrated the strongest correlation with a decrease in CFR, as the reduction rates increased by 0.67% for every additional percentage point of booster vaccination rates.

**Table 4.** The multiple linear regression analysis between reduction rates of CFRs and influencing factors

Category	Variable	Model 1^a^		Model 2^b^		Model 3^c^	
		*β*	95%CI	*β*	95%CI	*β*	95%CI
Public health interventions	Changes in total tests per thousand people	–	–	–	–	−0.01	(−0.02, 0.01)
	Changes in stringency index	0.37	(−1.14, 1.89)	–	–	–	–
	Changes in fully vaccine rates (%)	0.89	(−0.34, 2.11)	–	–	–	–
	Booster vaccine rates (%)	–	–	0.60*	(0.11, 1.09)	0.67*	(0.09, 1.24)
Social and economic characteristics of the country	Population density	0.19*	(0.05, 0.33)	0.05	(−0.00, 0.11)	0.00	(−0.06, 0.06)
	GDP	–	–	0.00	(−0.00, −0.00)	–	–
	Hospital beds per thousand people	3.28	(−2.33, 8.89)	4.15*	(1.41, 6.89)	2.45	(−0.54, 5.44)
	Human development index	–	–	–	–	−136.50	(−274.23, 1.23)
Demographic characteristics	The proportion of individuals over 65 years old (%)	–	–	–	–	–	–
	Diabetes prevalence (%)	−4.90	(−14.31, 4.51)	0.50	(−2.95, 3.94)	−1.42	(−5.80, 2.95)
	Obesity prevalence (%)	4.17	(−2.31, 10.65)	–	–	1.80	(−0.75, 4.35)
	Smoking rates (%)	−4.66*	(−8.98, −0.33)	−1.59	(−3.47, 0.29)	–	–

a The multiple linear regression analysis between reduction 1 and influencing factors.

b The multiple linear regression analysis between reduction 2 and influencing factors.

c The multiple linear regression analysis between reduction 3 and influencing factors.

* *P* < 0.05.

### Sensitivity analysis

The findings were consistent when each wave was extended by 14 days. The results of the sensitivity analysis are available in the Supplementary Materials (Supplementary Table S1).

## Discussion

The emergence of COVID-19 has brought catastrophic harm to human beings [18]. WHO published the 2023–2025 COVID-19 Strategic Preparedness and Response Plan to guide countries in transitioning to long-term management of COVID-19 as an ongoing health issue [19]; however, gaps remain in our understanding of the —temporal–spatial patterns of COVID-19 CFRs across countries. Studying the temporal and spatial epidemic characteristics of COVID-19 CFRs and their influencing factors is critical for decision-making and optimizing health resources for COVID-19. Our results found that COVID-19 CFRs varied across different continents and countries during three waves, and the reduction rates of CFR were significantly associated with population density, smoking rates, hospital beds per thousand, and booster vaccination.

Studies also suggested that there were significant variations in CFRs across different epidemic waves [20, 21], which was consistent with our findings. On the one hand, this may be related to different dominant variants, as the transmission intensity and the severity of different variants were diverse [22]. For instance, the transmissibility of the Alpha variant was 43–82% higher than the original strain, but no apparent alteration in disease severity was observed, while Beta and Gamma resulted in an escalation of mortality [17]. It’s worth spotting the light on the Omicron variant, which was initially reported in November 2021. Due to Omicron’s high transmissibility but low fatality rate [6], COVID-19 cases increased significantly while deaths decreased significantly in Wave 3 compared to the previous two waves.

Currently, vaccination is widely recognized as a crucial public health measure in fighting against COVID-19, effectively reducing the fatality rate [7]. Our findings indicate a positive correlation between higher booster vaccination rates and decreased CFR rates. Specifically, in comparison to Wave 1, the reduction rates of CFR increased by 0.60% for every additional percent of booster vaccination rate in Wave 3. When compared to CFRs in Wave 2, it was the main factor contributing to the reduction rates in Wave 3. Evidence suggests that the effectiveness of the COVID-19 vaccine may diminish over time [23], which could potentially explain this phenomenon. The booster dose played a crucial role in enhancing the efficacy of vaccination and managing the spread of the virus [24].

Our findings indicated a positive correlation between population density and the reduction rates of CFR in Wave 2 when compared to the CFRs observed in Wave 1. Consistent with our study, previous studies have shown that COVID-19 outbreaks have disproportionately affected cities with high population density [8], and a significant association has been established between COVID-19 CFR and population density (r = 0.81) [25].

With regard to medical resources, compared with Wave 1, the reduction rate of CFR was increased by 4.14% for each additional hospital bed per thousand people in Wave 3. Ergonül et al. also found the increased number of hospital beds had a significant effect on decreasing the national CFR [8]. This could be linked to the substantial recovery rate of COVID-19 patients in regions with sufficient medical and health resources [26]. Besides, the CFR of COVID-19 may also be associated with the improvement of medical treatment levels, due to the continuous exploration and improvement of relevant protection technologies and treatment methods for patients [27].

We observed a negative association between smoking rates and the reduction rates of CFR. Similarly, Clift et al. found that a higher number of cigarettes smoked per day was associated with a higher risk of COVID-19 death (OR: 10.02, 95%CI: 2.53–39.72) in a study using UK Biobank data (N = 421,469) [12]. Smoking could impair mucociliary oscillation and reduce cough reflex sensitivity, further impairing the respiratory barrier, which would possibly explain this phenomenon [28].

Our study demonstrated the temporal and spatial differences between COVID-19 CFRs at the global level. Including 30 major countries attacked in Wave 1, we longitudinally compared the reduction rates of CFR and potential influencing factors during the epidemic periods of different COVID-19 variants, providing evidence for the prevention and control of the COVID-19 epidemic. Furthermore, the influencing factors included in this study were relatively comprehensive, which could better reflect the impact of various factors on COVID-19 CFRs.

However, several limitations should be mentioned for our study. First, the main outcome of our study is CFR, but it may be overestimated or underestimated due to asymptomatic cases or distinct definitions and reporting standards in different countries [29]. Second, the stable setting of certain indicators throughout the study period may impact the demographic base during varying epidemic peaks. In the future, the latest data are needed to estimate the association between the CFRs and these factors more accurately. Third, with the development of the epidemic, the association between CFR and possible influencing factors may not be strong enough due to the influence of survival bias and peak incidence rate or infection, therefore the extrapolation of results should be done with caution. Fourth, there may be a lag effect on vaccination [30]. For the reason that the booster vaccine was not widely received during the period we studied, further investigation is needed considerably to analyse the impact of booster vaccinations on COVID-19 CFRs more specifically. Last but not least, not only vaccines but also previous infections could produce antibodies. However, without access to the COVID-19 infection history data, the results of this study should be interpreted with caution. Taking COVID-19 infection history into consideration could be very helpful in assessing the effect of vaccines more precisely.

## Conclusion

Based on the analysis of CFRs and potential influencing factors in 30 countries during three waves, our study revealed significant variations in COVID-19 CFRs among countries, and the reduction rates of CFRs between different waves were associated with booster vaccine, population density, hospital beds per thousand people, and smoking rates. These insights could provide valuable information for the prevention and control of COVID-19.

## Supporting information

Zhao et al. supplementary material Zhao et al. supplementary material

## Data Availability

All data used in this analysis were collected from online open datasets.

## Abbreviations

CFR: case fatality rate
COVID-19: Coronavirus disease 2019
SARS-CoV-2: severe acute respiratory syndrome coronavirus 2
SD: standard deviation
VOC: variant of concern

## Supplementary material

For supplementary material accompanying this paper visit http://doi.org/10.1017/S0950268824000852. click here to view supplementary material
