# Supplementary material for: The spatial–temporal variations and influencing factors of COVID-19 case fatality rate: a worldwide study in 30 countries from February 2021 to May 2022
Source: Epidemiol Infect. 2024 Oct 17;152:e124. doi: 10.1017/S0950268824000852 (PMC11502459; doi:10.1017/S0950268824000852)
Supplement: Zhao et al. supplementary material [file S0950268824000852sup001.docx]

**Supplementary materials**

**Table S1.** The multiple linear regression analysis of the three reduction rates of COVID-19 CFRs and potential influencing factors in the sensitivity analysis

**Figure S1.** Cases and deaths of COVID-19 in the world from February 22, 2020 to June 22, 2022

**Figure S2.** Cases and deaths of COVID-19 in 30 countries from January 22, 2020 to June 22, 2022

**Figure S3.** The correlation analysis of Reduction 1 and influencing factors

**Figure S4.** The correlation analysis of Reduction 2 and influencing factors

**Figure S5.** The correlation analysis of Reduction 3 and influencing factors

**Figure S6.** The forest plots of COVID-19 CFRs for 30 countries in Wave 1

**Figure S7.** The forest plots of COVID-19 CFRs for 30 countries in Wave 2

**Figure S8.** The forest plots of COVID-19 CFRs for 30 countries in Wave 3

**Figure S9.** The forest plots of COVID-19 CFRs for 30 countries grouped by continent in Wave 1

**Figure S10.** The forest plots of COVID-19 CFRs for 30 countries grouped by continent in Wave 2

**Figure S11.** The forest plots of COVID-19 CFRs for 30 countries grouped by continent in Wave 3

| **Table S1.** The multiple linear regression analysis of the three reduction rates of COVID-19 CFRs and potential influencing factors in the sensitivity analysis | | | | | | | | | |
| --- | --- | --- | --- | --- | --- | --- | --- | --- | --- |
| **Category** | **Variable** | **Model 1^a^** | |  | **Model 2^b^** | |  | **Model 3^c^** | |
|  |  | ***β*** | ***95%CI*** |  | ***β*** | ***95%CI*** |  | ***β*** | ***95%CI*** |
| **Public health interventions** | Changes in total tests per thousand people | -- | -- |  | -- | -- |  | 0.00 | (-0.02, 0.01) |
|  | Changes in stringency index | 0.45 | （-0.90, 1.80） |  | -- | -- |  | -- | -- |
|  | Changes in fully vaccinated rates (%) | 1.10 | （-0.018, 2.21） |  | -- | -- |  | -- | -- |
|  | Booster vaccine rates (%) | -- | -- |  | 0.75^*^ | （0.18, 1.31） |  | 0.72^*^ | (0.08, 1.37) |
|  |  |  |  |  |  |  |  |  |  |
| **Social and economic characteristics of the country** | Population density | 0.18^*^ | （0.06, 0.30） |  | 0.05 | （0.00, 0.11） |  | 0.00 | (-0.06, 0.06) |
|  | GDP | -- | -- |  | 0.00 | （-0.00, 0.00） |  | -- | -- |
|  | Hospital beds per thousand people | 2.86 | （-2.29, 8.00） |  | 4.05^*^ | （1.35, 6.74） |  | 2.84 | (-0.20, 5.89) |
|  | Human development index | -- | -- |  | -- | -- |  | -148.00 | (-304.27, 8.27) |
|  |  |  |  |  |  |  |  |  |  |
| **Demographic characteristics** | The proportion of individuals over 65 years old (%) | -- | -- |  | -- | -- |  | -- | -- |
|  | Diabetes prevalence (%) | -5.07 | （-11.24, 1.10） |  | 0.31 | （-2.33, 2.95） |  | -0.02 | (-3.11, 3.07) |
|  | Obesity prevalence (%) | 4.24 | （-1.10, 9.58） |  | -- | -- |  | 1.22 | (-1.21, 3.64) |
|  | Smoking rates (%) | -4.63^*^ | （-8.24, -1.02） |  | -1.09 | （-3.06, 0.89） |  | -- | -- |

^*^*P*<0.05.

^a^ The multiple linear regression analysis between reduction 1 and influencing factors.

^b^ The multiple linear regression analysis between reduction 2 and influencing factors.

^c^ The multiple linear regression analysis between reduction 3 and influencing factors.


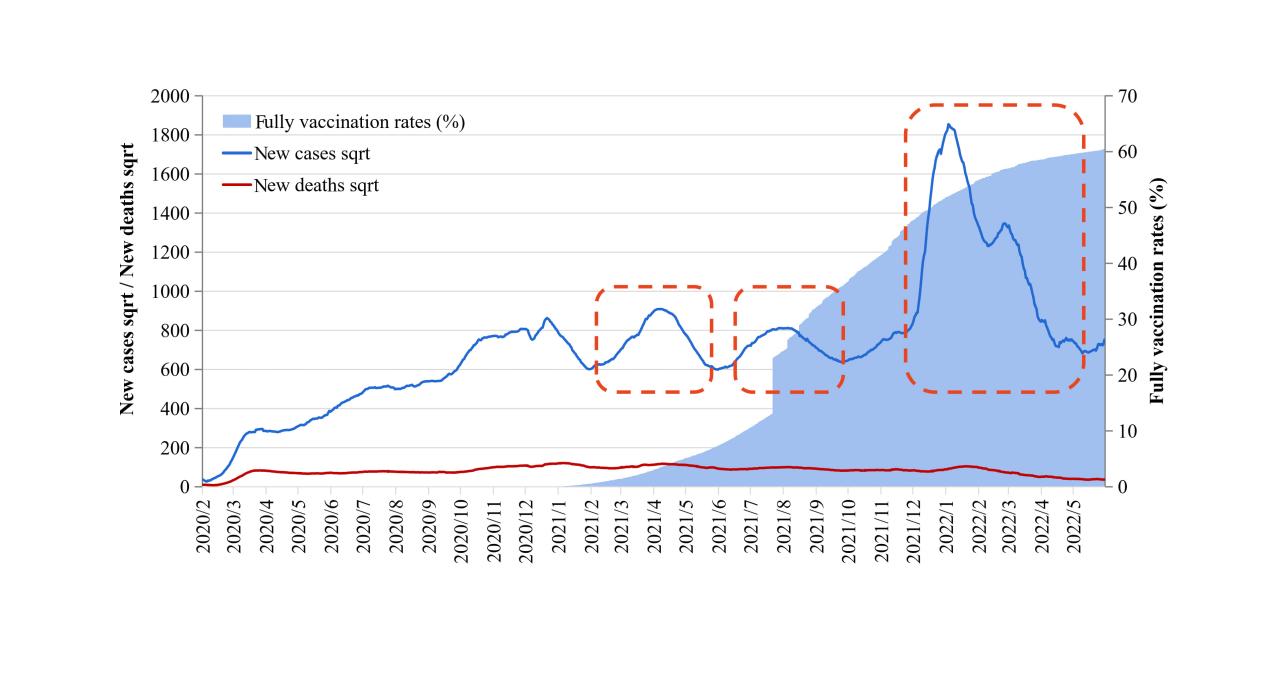


**Figure S1.** Cases and deaths of COVID-19 in the world from February 22, 2020 to June 22, 2022


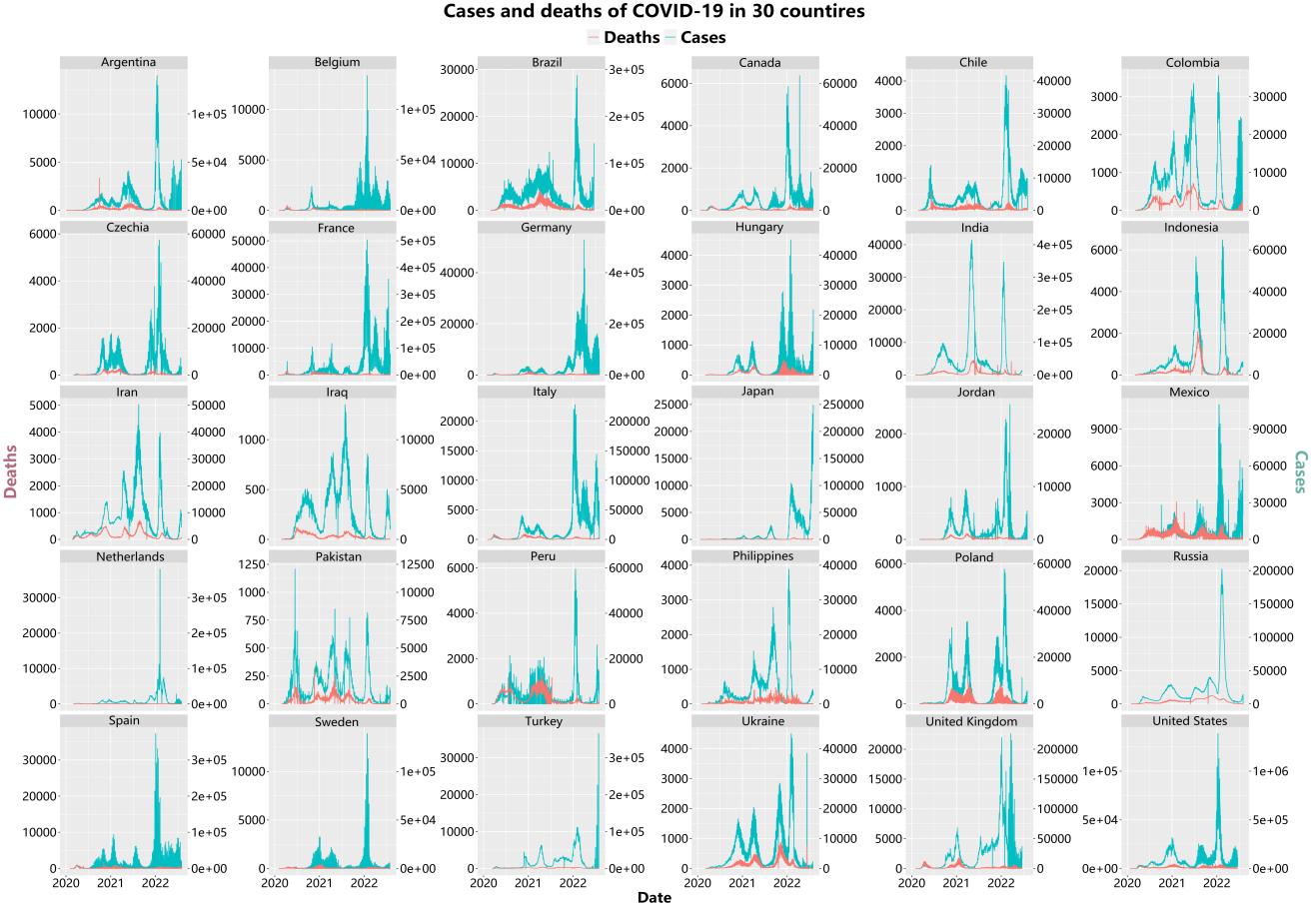
Figure S2. Cases and deaths of COVID-19 in 30 countries from January 22, 2020 to June 22, 2022


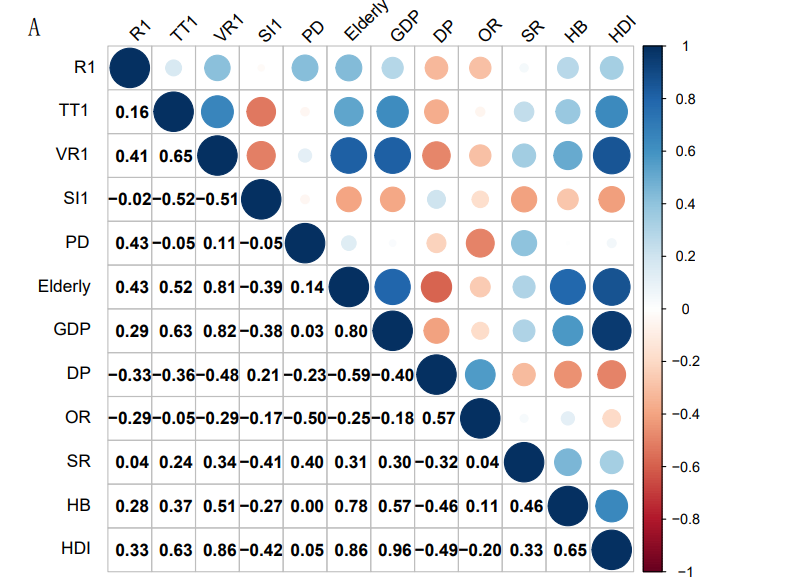


**Figure S3.** The correlation analysis of Reduction 1 and influencing factors.

_R1, the reduction rates of COVID-19 CFRs in Wave 2 compared with the CFRs in Wave 1; TT1, the changes in total tests per thousand people in Wave 2 when compared with Wave 1; VR1, the changes in fully vaccinated rates in Wave 2 when compared with Wave 1; SI1, the changes in stringency index in Wave 2 when compared with Wave 1; PD, population density; Elderly, the proportion of individuals over 65 years old; GDP, gross domestic product; DP, diabetes prevalence; OR, obesity rates; SR, smoking rates; HB, hospital beds per thousand people; HDI, human development index._


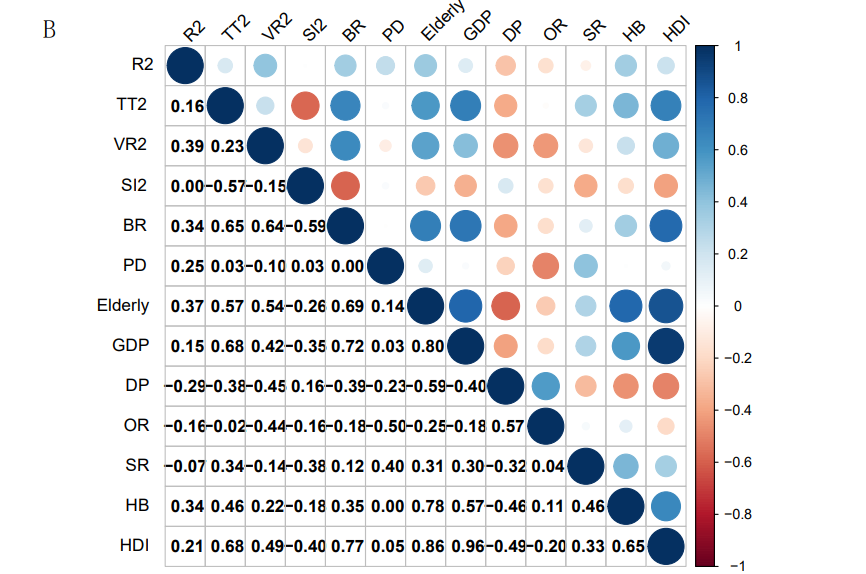


**Figure S4.** The correlation analysis of Reduction 2 and influencing factors.

_R2, the reduction rates of COVID-19 CFRs in Wave 3 compared with the CFRs in Wave 1; TT2, the changes in total tests per thousand people in Wave 3 when compared with Wave 1; VR2, the changes in fully vaccinated rates in Wave 3 when compared with Wave 1; SI2, the changes in stringency index in Wave 3 when compared with Wave 1; BR, COVID-19 boosters administered per 100 people in Wave 3; PD, population density; Elderly, the proportion of individuals over 65 years old; GDP, gross domestic product; DP, diabetes prevalence; OR, obesity rates; SR, smoking rates; HB, hospital beds per thousand people; HDI, human development index._


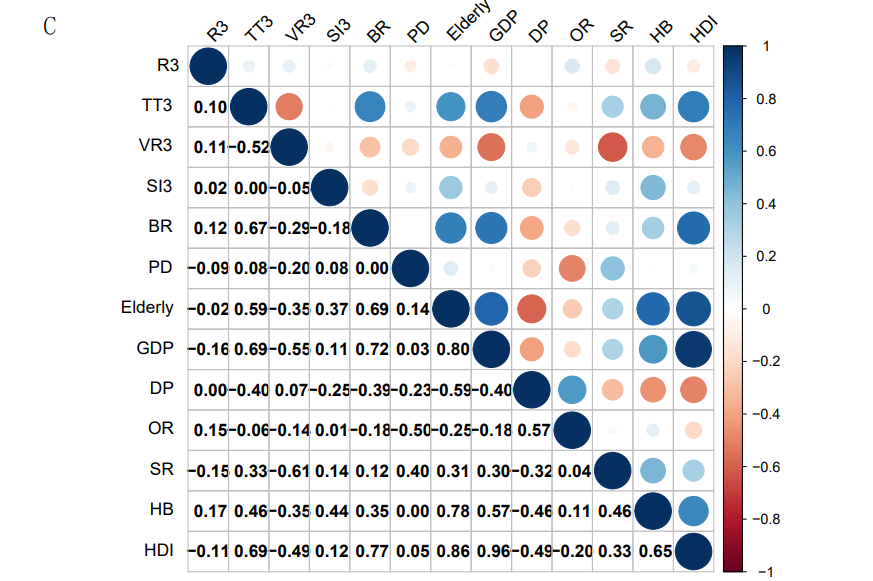


**Figure S5.** The correlation analysis of Reduction 3 and influencing factors

_R3, the reduction rates of COVID-19 CFRs in Wave 3 compared with the CFRs in Wave 2; TT3, the changes in total tests per thousand people in Wave 3 when compared with Wave 2; VR3, the changes in fully vaccinated rates in Wave 3 when compared with Wave 2; SI3, the changes in stringency index in Wave 3 when compared with Wave 2; BR, COVID-19 boosters administered per 100 people in Wave 3; PD, population density; Elderly, the proportion of individuals over 65 years old; GDP, gross domestic product; DP, diabetes prevalence; OR, obesity rates; SR, smoking rates; HB, hospital beds per thousand people; HDI, human development index._


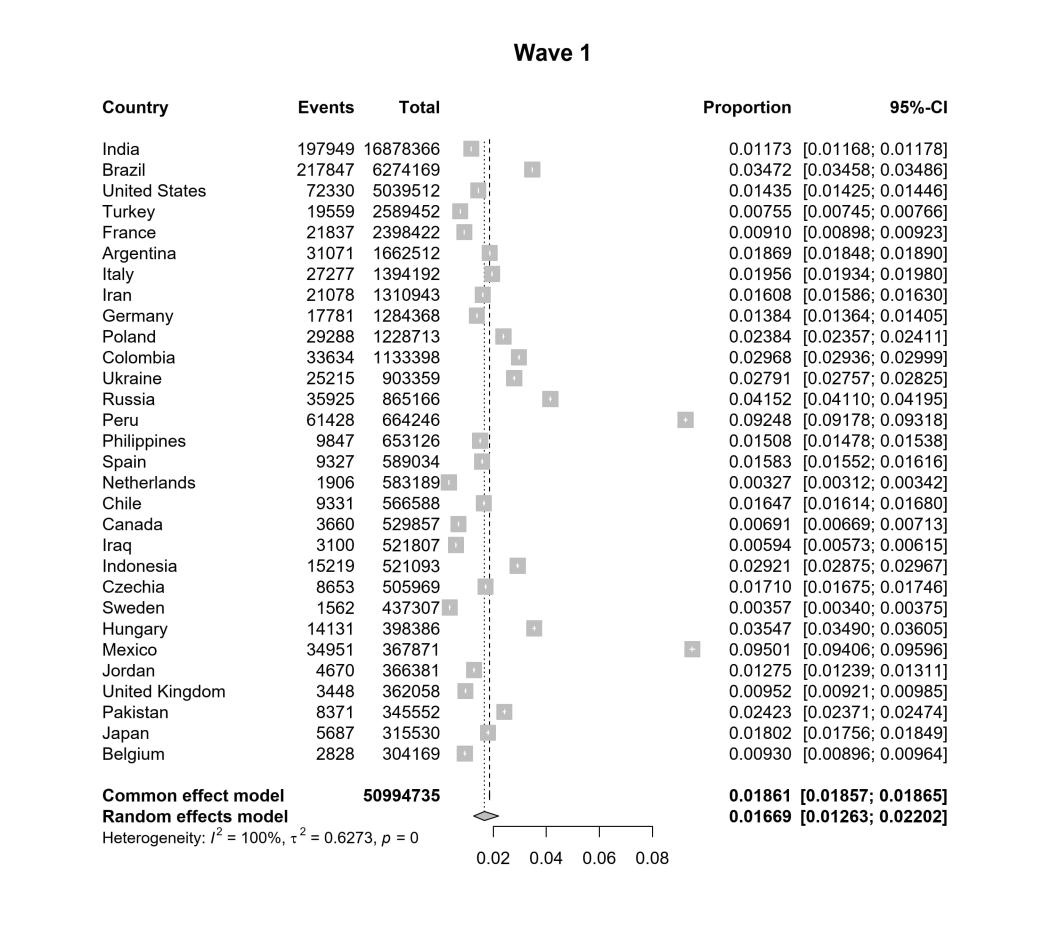


**Figure S6.** The forest plots of COVID-19 CFRs for 30 countries in Wave 1


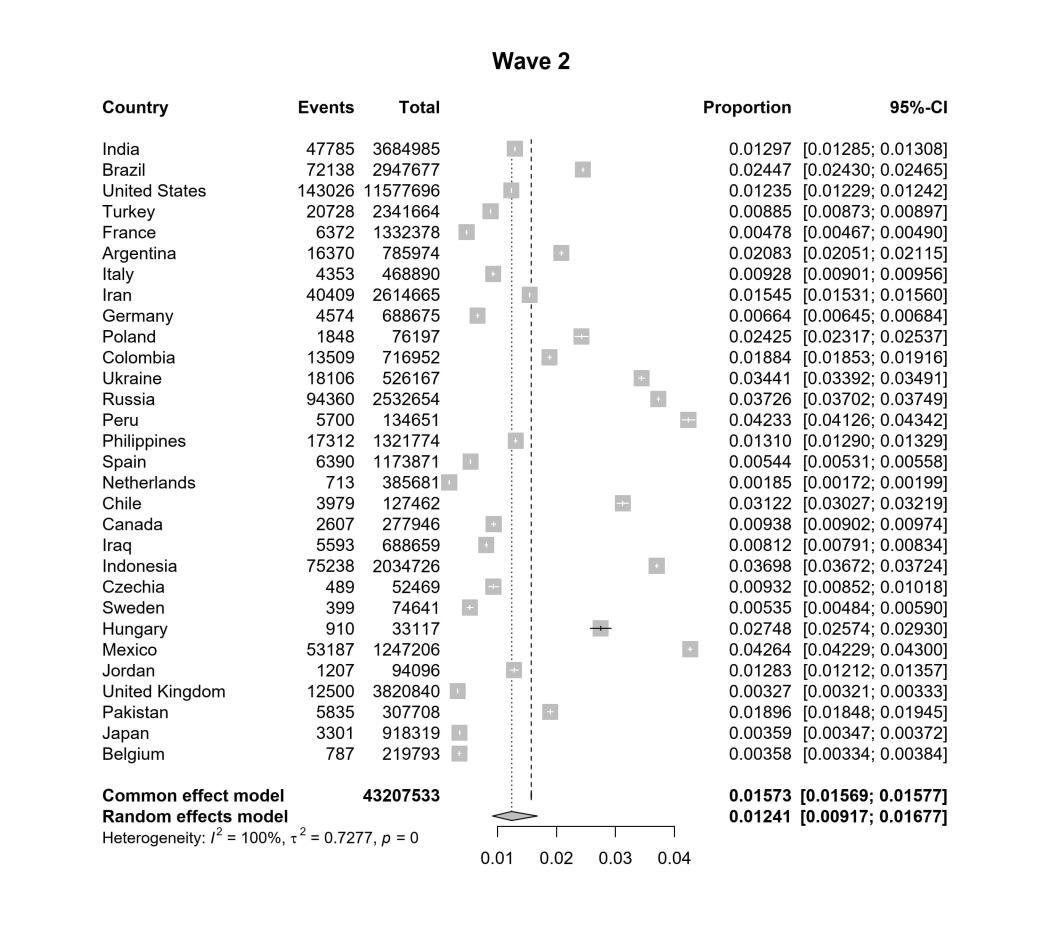


**Figure S7.** The forest plots of COVID-19 CFRs for 30 countries in Wave 2


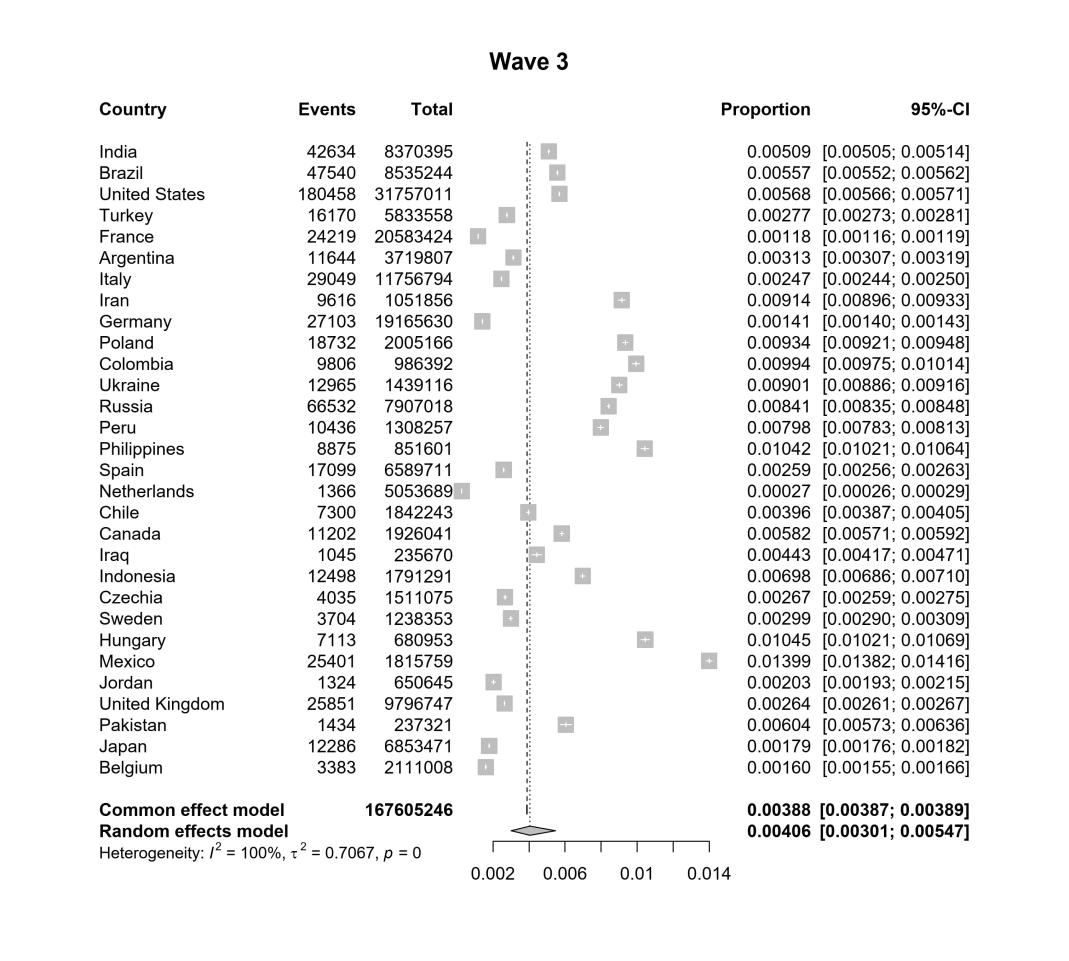


**Figure S8.** The forest plots of COVID-19 CFRs for 30 countries in Wave 3


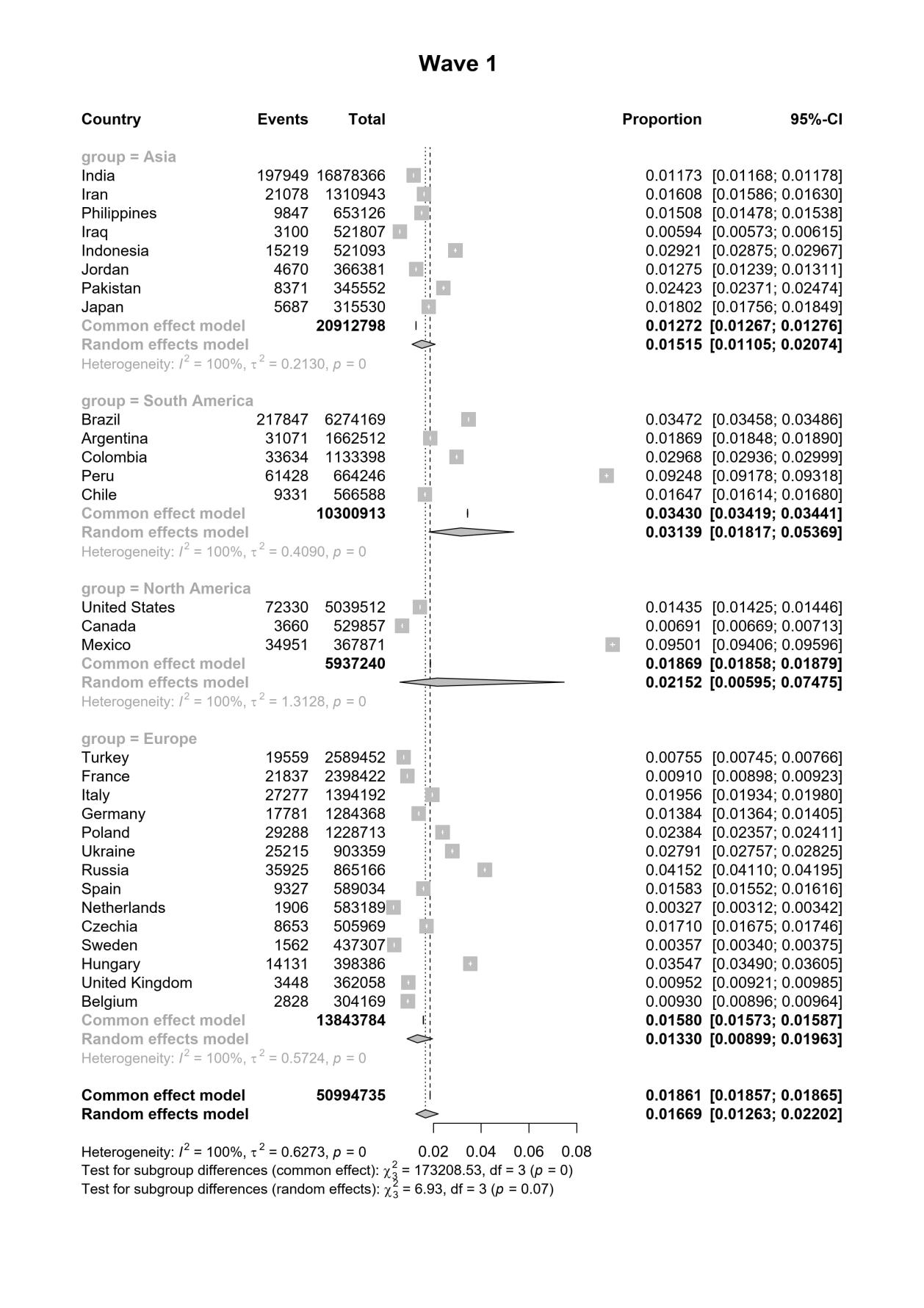


**Figure S9.** The forest plots of COVID-19 CFRs for 30 countries grouped by continent in Wave 1


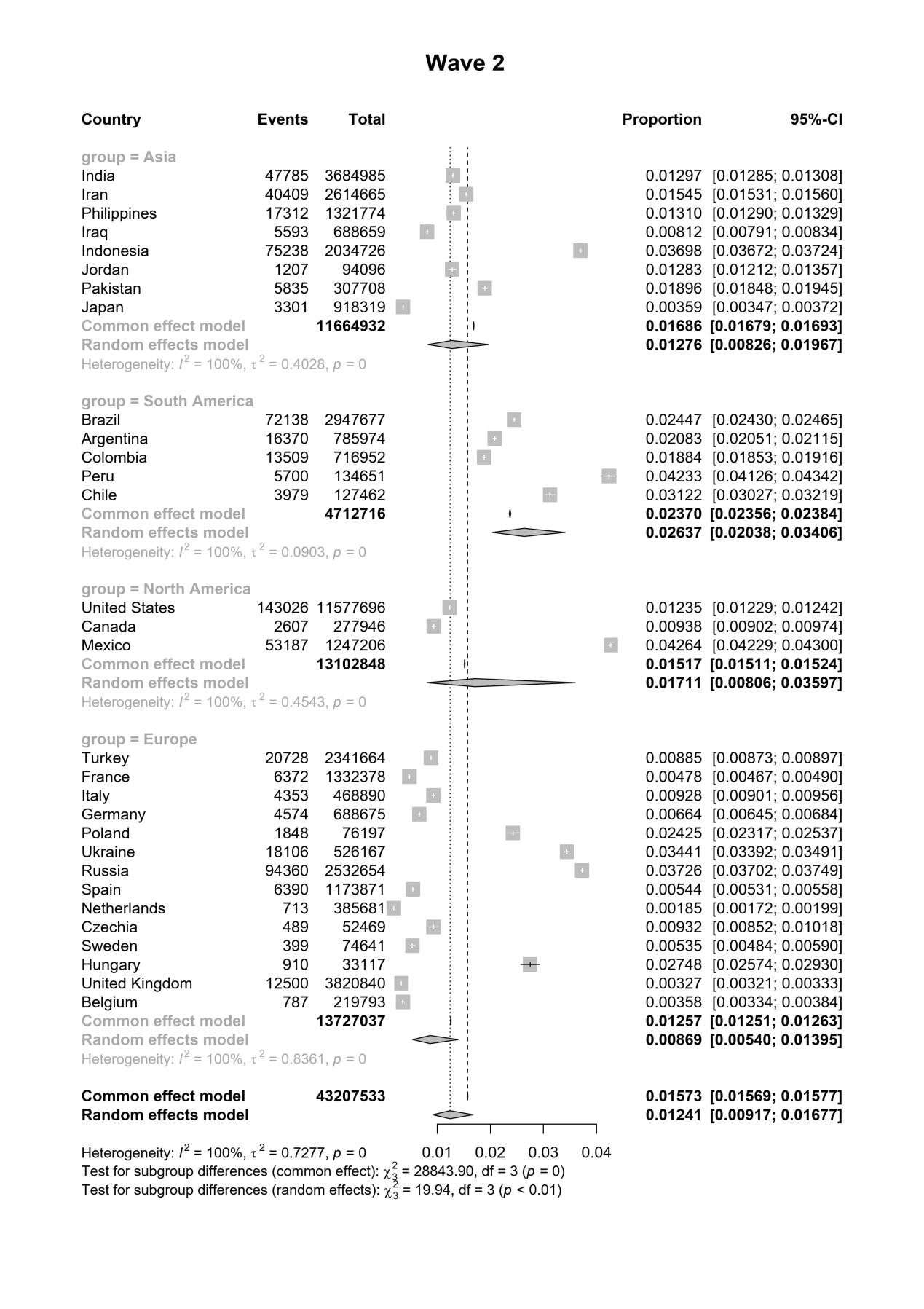


**Figure S10.** The forest plots of COVID-19 CFRs for 30 countries grouped by continent in Wave 2


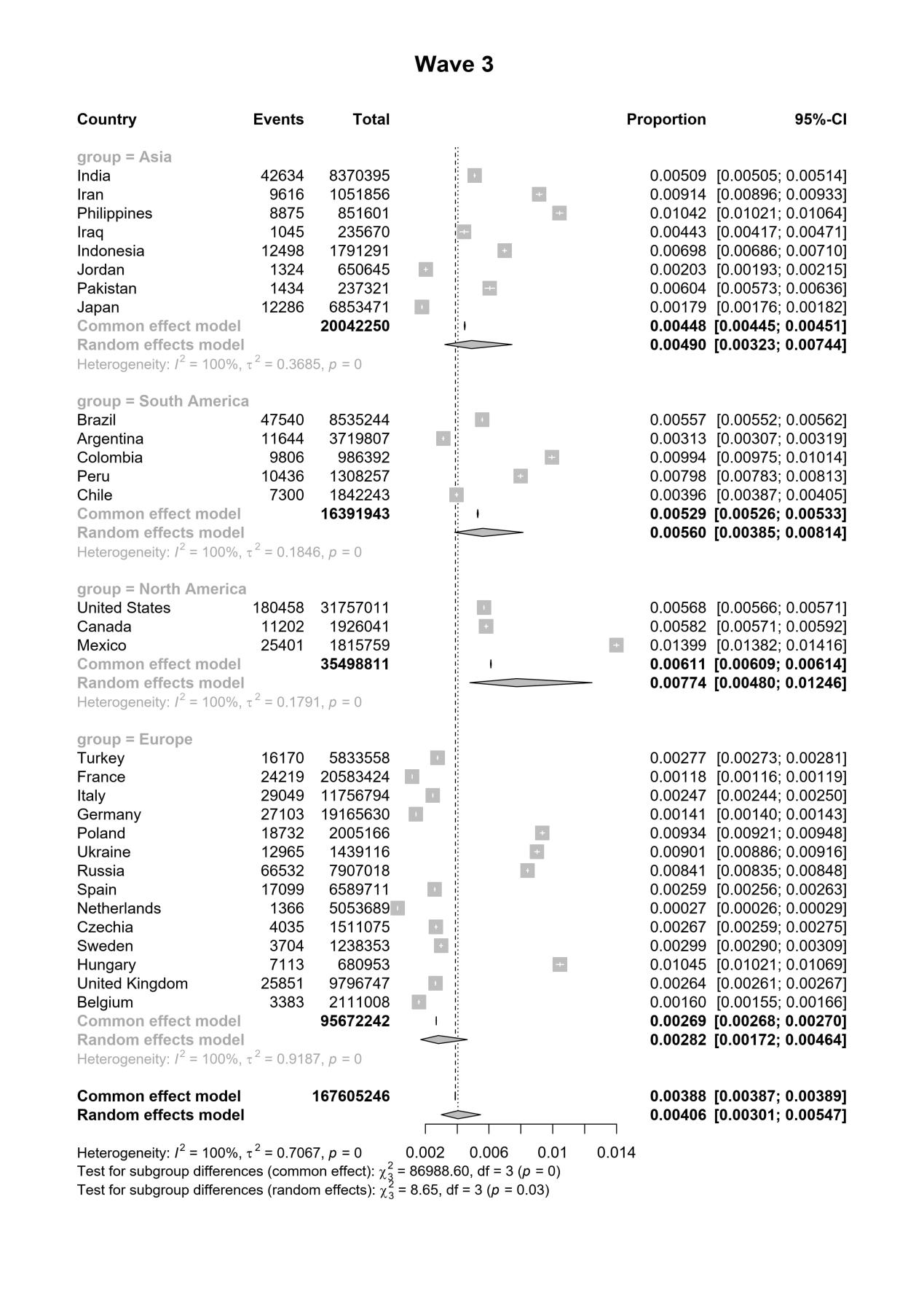


**Figure S11.** The forest plots of COVID-19 CFRs for 30 countries grouped by continent in Wave 3
